# WT1 gene expression as a prognostic marker in advanced serous epithelial ovarian carcinoma: an immunohistochemical study

**DOI:** 10.1186/1471-2407-6-90

**Published:** 2006-04-11

**Authors:** Wirote Netinatsunthorn, Jitti Hanprasertpong, Chavaboon Dechsukhum, Roengsak Leetanaporn, Alan Geater

**Affiliations:** 1 Department of Obstetrics and Gynecology, Faculty of Medicine, Prince of Songkla University, Hatyai, Songkhla, Thailand; 2 Department of Pathology, Faculty of Medicine, Prince of Songkla University, Hatyai, Songkhla, Thailand; 3 Epidemiology Unit, Faculty of Medicine, Prince of Songkla University, Hatyai, Songkhla, Thailand

## Abstract

**Background:**

WT1 is a tumor suppressor gene responsible for Wilms' tumor. WT1 reactivity is limited to ovarian serous carcinomas. Recent studies have shown that WT1 plays an important role in the progression of disease and indicates a poorer prognosis of human malignancies such as acute myeloid leukemia and breast cancer. The aims of this study were to determine the survival and recurrence-free survival of women with advanced serous epithelial ovarian carcinoma in relation to WT1 gene expression.

**Methods:**

The study accrued women over an 18-year period, from 1987–2004. During the study period, 163 patients were diagnosed with advanced serous epithelial ovarian carcinoma and had undergone complete post-operative chemotherapy, but the final study group comprised 99 patients. The records of these women were reviewed and the paraffin-embedded tissue of these women stained with WT1 immunostaining. Survival analysis was performed using Kaplan-Meier and Cox regression methods.

**Results:**

Fifty patients showed WT1 staining and forty-nine did not. Five-year survival of non-staining and staining groups were 39.4% and 10.7% (p < 0.00005); five-year recurrence-free survival of these groups were 29.8% and ≤ 7.5% (p < 0.00005), respectively. For survival the HR of WT1 staining, adjusted for residual tumor and chemotherapy response, was 1.98 (95% CI 1.28–3.79), and for recurrence-free survival the HR was 3.36 (95% CI 1.60–7.03). The HR for recurrence-free survival was not confounded by any other variables.

**Conclusion:**

This study suggests that expression of WT1 gene may be indicative of an unfavorable prognosis in patients with advanced serous epithelial ovarian carcinoma.

## Background

Ovarian cancer is one of the leading problems in gynecologic malignancy in females worldwide. In Thailand, ovarian cancer is the sixth most common cancer among females, with approximately 1,655 new cases per year [1]. Although several histologic types of epithelial ovarian cancer exist, approximately 60% are of the serous epithelial type [2]. Due to their nonspecific initial symptoms, 70% of patients have widespread metastatic disease at the time of diagnosis [3]. Survival rate is generally low. Despite advances in evaluation and treatment, the survival rate for all stage of ovarian cancer has remained constant over the past 30 years [4]. Parameters such as International Federation of Gynecology and Obstetrics (FIGO) stage at diagnosis, histologic grade, cell type, and amount of residual disease after first surgery, represent the most important factors to date for distinguishing between those patients who will have favorable and unfavorable clinical outcomes. In addition, more recent various immunohistochemical studies of ovarian cancer have suggested that expression of particular markers may help in predicting outcome, and therefore guide therapeutic choices [2,4-8].

Wilms' tumor is a kidney malignancy of childhood that is thought to arise as a result of inactivation of both alles of the Wilms' tumor gene (WT1) located at chromosome 11p13 [9,10]. WT1 is a tumor suppressor gene responsible for Wilms' tumor. In normal human tissue, WT1 is restricted to kidney, testis, ovary, spleen, hematopoietic precursors, and the meseothelial cell lining of visceral organs [11].

Recent studies have shown that WT1 plays an important role in the progression of disease and prognosis of human malignancies [12-15]. Bergmann et al. reported that high expression of WT1 mRNA is associated with a worse long-term prognosis of acute myeloid leukemias patients [13]. Similarity, Miyoshi et al. reported that measurement of WT1 mRNA levels in tumor tissues might be useful as a new prognostic factor in breast cancer patients [15]. In epithelial ovarian tumors, WT1 expression has been detected [16]. WT1 reactivity is limited to ovarian serous carcinomas, and is not found in mucinous carcinomas [18,19]. However, until now, there are no reports of association between survival and the WT1 gene. The aim of this study, therefore, was to determine the survival and recurrence-free survival of women with advanced serous epithelial ovarian carcinoma in relation to WT1 gene expression. In the present study, expression of WT1 was examined by immunohistochemistry.

## Methods

### Patients

This study was approved by the Research Committee of the Faculty of Medicine, Prince of Songkla University. The medical records of all women diagnosed with FIGO stage III and IV serous ovarian carcinoma in Department of Obstetrics and Gynecology, Songklanagarind Hospital, from January 1987 to December 2004, were retrospectively reviewed. The clinicopathology details such as age, parity, chief complaint, FIGO stage, histologic grade, residual tumor, and chemotherapy response were assessed. The standard operation procedures were total abdominal hysterectomy, bilateral adnexectomy, omentectomy, lymph node sampling, and peritoneal washing. After tumor debulking these women received adjuvant chemotherapy, platinum analog and cyclophosphamide, for six cycles. For women who underwent only tumor biopsy at the first operation, chemotherapy was given for three cycles, then interval debulking of the tumor performed in the same fashion, followed by an additional three cycles of chemotherapy. The tissues from these patients were used for the study group. Fully informed consent was obtained from all patients prior to surgery, and tumor samples were collected during surgery. The response of the women to chemotherapy was evaluated use WHO criteria [20]. All deaths were registered by the Medical Statistic Unit and Cancer Registry Unit of Songklanagarind Hospital, and the Department of Provincial Administration, Ministry of Interior, according to death certificates issued by a physician stating the cause of death. The status of all living and/or lost to follow-up patients was confirmed directly by telephoning or mailing and by checking the census records from the Hatyai City Municipality.

### Immunohistochemical staining

The hematoxylin and eosin stained histologic slides from each patient were reviewed and the corresponding paraffin-embedded tissue blocks selected by one pathologist of the Department of Pathology, Prince of Songkla University. Sections (5μm) from archival formalin-embedded tissue blocks were mounted on the salinized slides, and baked overnight at 60°C. They were then deparaffinized in xylene, rehydrated in a decreasing ethanol series and rinsed in phosphate buffered saline (PBS), pH 7.4. Endogeneous peroxidase was blocked using 0.3% H_2_O_2 _in PBS for 5 min. The tissue slides were then subjected to antigen retrieval by microwave followed by blocking with 10% normal horse serum. The tissue slides were then incubated with monoclonal mouse antibody against WT1 (1:100) overnight at 4°C followed by addition of biotinylated rabbit anti-mouse IgG secondary antibody (Vecta staining kit) and then avidin-biotin-peroxidase complex (Vecta staining kit) according to the manufacturer's protocol. Visualization of the immunoreaction was achieved using diaminobenzidine tetrahydrochloride and hydrogen peroxide (0.03% in 50 mM Tris-HCl, pH 7.6) for 10 min. Sections were rinsed in tap water, counterstained with hematoxylin, dehydrated through graded alcohol, cleared in xylene, and mounted using DPX. Positive nuclear immunostaining in Sertoli cells had to be demonstrated in the immunohistochemical study for the quality control purpose.

### Determination of staining

**Figure 1:**
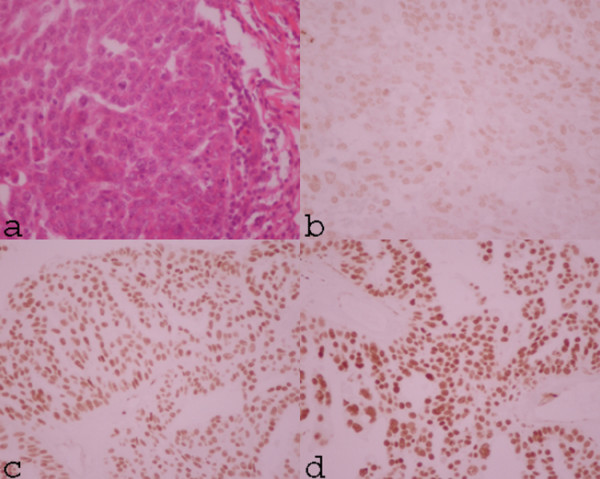
The H&E sections show a solid area of tumor which consists of cuboidal tumor cells with eosinophilic cytoplasm and vesicular nuclei, with prominent nucleoli. Mitotic figures are frequently observed (a). The immunohistochemical study of the same patient shows faint nuclei staining (intensity 1+) for WT1 protein in the most of tumor cells (b). The immunohistochemical studies show strong immunoreactivity in the nuclei in the majority of tumor cells (c: intensity 2+, d: intensity 3+)

The immunostained slides were examined using an Olympus light microscope equipped with a grid eyepiece. The sections were evaluated by one pathologist. The area of tumor cells was screened and a minimum of 1,000 tumor cells were counted for each slide, excluding foci of inflammation and necrosis. The number of tumour cells with nuclear staining was recorded and reported as percentage staining and the intensity classified as 0, 1+, 2+, 3+ (Fig. 1) [16].

### Statistical analysis

Survival time was calculated from the date of starting treatment to the date of death or censoring at the last followed-up. Similarly, recurrence-free survival was calculated from the date of starting treatment to the date of recurrence or death or last follow-up, whichever occurred first, confining the analysis to those patients who showed a complete response to chemotherapy.

Patients were divided into two groups, those with percentage staining ≥ median and those with percentage staining < median. Survival profiles of the entire groups were examined using Kaplan-Meier plots. Multivariate Cox proportional hazards regression was used to examine the crude and adjusted hazard ratios for WT1 staining and to identify these variables with an independent association with survival and with recurrence-free survival. For multivariate modeling, all variables were initially included and the least significant variable was removed in a stepwise process until all remaining variables showed a statistically significant contribution (P < 0.05) to the fit of the model. WT1 staining was retained irrespective of its statistical significance. Statistical significance of each variable was assessed using the change in log-likelihood of successive models.

### Power of the study

The study accrued women over an 18-year period, from 1987–2004. During the study period, 163 patients were diagnosed with advanced serous epithelial ovarian carcinoma and had undergone complete post-operative chemotherapy. Among these, 36 had no medical records and another 28 had no tumour specimens. Thus, the final study group comprised 99 patients. While there are no previous studies of WT1 gene and survival times of ovarian cancer, the 5-year overall survival of advanced epithelial ovarian carcinoma has been reported to be between 11 and 41% [17]. For the purposes of power calculation, we assumed a 5-year overall survival of about 23%, corresponding to a rate of death of 0.293 year^-1^, based on an assumed exponential survival profile. We planned to compare the survival of patients showing WT1 staining percentage ≥ median with that of patients have < median percentage staining. Assuming the rate to be equally increased and decreased among the ≥ median group with and < median group, then to detect a difference in 5-year survival of 14% (death rate = 0.393 year^-1^) and 36% (death rate = 0.204 year^-1^) respectively in the two groups as significant at a 2-sided alpha of 0.05 with a power of 80% required 99 subjects.

## Results

Mean age at diagnosis was 54.53 years (range 18–80 years). The most prominent presenting symptom were abdominal distension (54.5%) and pelvic mass (33.3%). Most of the patients were in FIGO surgical stage III (85.9%). Only 50 patients (50.5%) were reactive for WT1. The distribution by WT1 staining intensity 1+, 2+, 3+ was 29.3%, 19.2%, 2.0%, respectively, and almost exactly half had no staining. Thus the patients were divided into non-staining and staining groups. The clinical outcome and other characteristics of the patients are shown in Table 1. The relationships of clinicopathologic variables and WT1 expression are shown in Table 2. No significant association between WT1 group and FIGO stage, histologic grade or residual tumor was found. Only chemotherapy response and residual tumor showed significant difference between WT1 staining and no WT1 staining patients.

**Figure 2:**
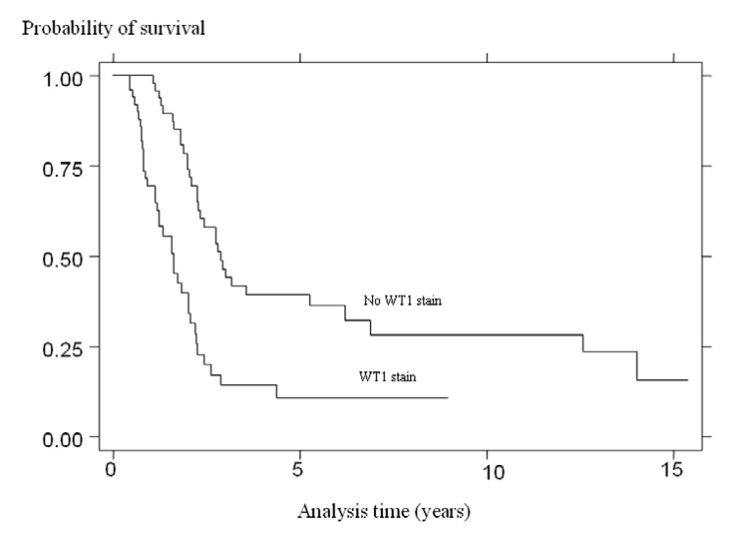
Kaplan-Meier overall survival profile of the 99 advanced serous epithelial ovarian carcinoma women

The 5-year survival of non-WT1 staining patients was 39.4% (95% CI 25.1 – 53.4) compared with 10.7% (95% CI 3.2 – 23.6) in the WT1-statining patients (p < 0.00005) (Fig. 2). In univariate Cox proportional hazards models, survival was found to be statistically significantly poorer in patients who did not respond to chemotherapy and in patients whose tissue showed WT1 staining. There was no evidence, however, that the hazard was increased with increasing percentage or increasing intensity of staining (Table 3). Both WT1 staining and chemotherapy response remained significant predictors of survival in a multivariate regression model. Stable and progressive disease were associated with hazard ratios of 2.29 (95% CI 0.80–6.13) and 2.93 (95% CI 1.53–5.62) compared with a complete response to chemotherapy, and WT1 staining with a hazard ratio of 1.98 (95% CI 1.28–3.79; p = 0.0138) (Table 4).

**Figure 3:**
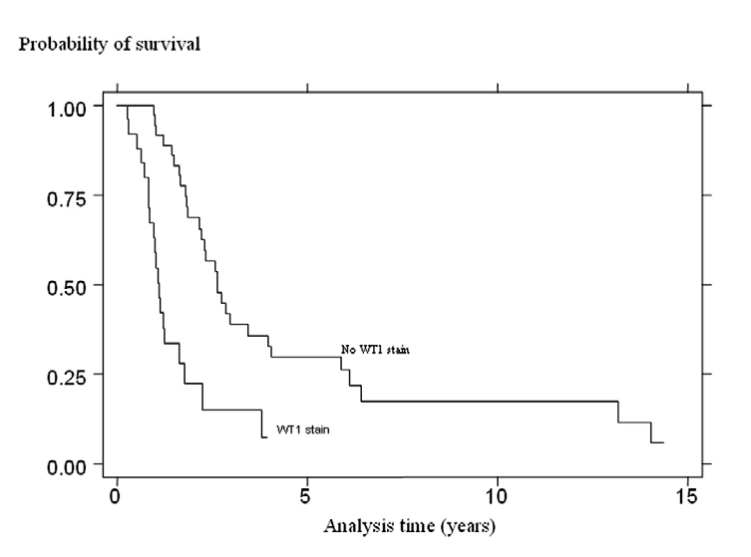
Kaplan-Meier recurrence-free survival profile of the 62 advanced serous epithelial ovarian carcinoma women with complete response to chemotherapy

Among patients who showed a complete response to chemotherapy, the 5-year recurrence-free survival of those who showed no WT1 staining was 29.8 percent (95% CI 15.6–45.4) compared with ≤ 7.5% (95% CI 0.6–27.2) in the WT1 statining patients (p < 0.00005) (Fig. 3). Cox proportional hazards modeling revealed WT1 staining to be the only statistically significantly predictor of recurrence-free survival, either in univariate models (Table 5) or after testing in a multivariate setting. The hazard ratio was 3.36 (95% CI 1.60–7.03; p = 0.0017) (Table 6). As with overall survival, there was no evidence that the hazard was increased with increasing percentage or increasing intensity of staining.

## Discussion

In the present study, we could demonstrate the immunohistochemical expression of WT1 gene in advanced serous ovarian carcinomas. There are many reports showing that in epithelial ovarian tumors, serous tumors generally revealed a high WT1 gene expression [16,18,19]. We found that about 50% of advanced serous ovarian carcinoma patients were reactive for WT1. Analogous findings have been recently reported by Schorge et al [21]. Conversely, Goldstein et al. reported that 93% of serous ovarian carcinoma patients were reactive for WT1 [18]. The reasons for these discrepancies are unclear. However this finding is unlikely to be due to the technical issue as we performed the strict quality control for the immunohistochemical study.

The molecular mechanism underlying the difference in the prevalence of WT1 geneexpression between our study and previous published data was not clear at the moment. Due to the fact that WT1 gene has been shown to be transcriptionally regulated by some upstream transcriptional factors like Sp1, PAX2, PAX8 and PEA3, it is worthwhile to further analyze the expression status of these upstream molecules in ovarian cancer specimens obtained from this population [22-26]. Moreover, as the biological function of WT1 has been shown to be influenced by other interactive proteins like p53 and par-4, the expression status of thesegenes should be evaluated in order to better define the prognostic value of WT1 gene expression [27,28]. This finding may reflect the different oncogenic pathway in this cancer in Thai population.

Since the report by Inoue et al. in 1994, it has become clear that WT1 gene expression is a new prognostic factor and new marker for the detection of minimal residual disease in acute leukemia [12]. The prognostic value of WT1 gene expression was corroborated by later studies in patients with acute leukemia, nephroblastoma and breast cancer [13-15,29,30].

In ovarian cancer, Shimizu et al. suggested that WT1 gene may be related to cell differentiation, and to the histologic subtypes of epithelial ovarian carcinomas [16]. Goldstein et al. reported that WT1 expression could be used as antibody to distinguish pancreatocobiliary and some ovarian epithelial neoplasms [18]. However, the relationship of WT1 expression in serous ovarian carcinoma with adverse outcome had not previously been reported. We found that WT1 gene expression and failure to respond to chemotherapy were significantly associated with overall survival and only WT1 gene expression to be significantly associated with recurrence-free survival. This is the first report that demonstrates the prognostic significance of WT1 gene expression in serous ovarian carcinoma. The impact of WT1 gene expression on the clinical outcome of this cancer was not demonstrated by previous studies [16,31]. The explanation for the discrepancy is not clear in the present time. However, the influence of genetic background could be one of responsible factors. Further study with larger sample size would be worthwhile to address this issue. These observations support the hypothesis that WT1 plays a role as an oncogene in this type of ovarian cancer, as has been demonstrated in leukemia and breast cancer [32].

Neither the quantitative level nor the intensity of WT1 expression were found to be related to overall or recurrence-free survival. By contrast, Garg et al. studied quantitative WT1 gene transcripts by competitive reverse transcription polymerase chain reaction in acute leukemia and found that accurate quantification of WT1 gene transcripts in both acute myeloid leukemia and acute lymphoblastic leukemia patients at presentation was an important prognostic pretreatment characteristic and a useful predictive marker of leukemia relapse [30].

In several previous studies, the prognostic value of other clinicopathologic variables (i.e. FIGO stage, histologic type, histologic grade, and primary residual tumor) has been shown [33,34], but the present study was unable to show any association with stage, histologic grade, or size of residual tumor. This difference might be explained by the fact that we enrolled only cases with advanced stage; at this stage earlier prognostic indicators might no longer have prognostic significance. The small number of stage IV patients also resulted in a wide confidence interval for the univariate hazard ratio for overall survival, extending upwards to over 2, so our data were not incompatible with FIGO stage as a prognostic indicator. Response to chemotherapy was a highly significant predictor of survival among our patients.

## Conclusion

Our study suggests that expression of WT1 gene may be indicative of an unfavorable prognosis in patients with advanced serous epithelial ovarian carcinoma.

## Competing interests

The author(s) declare that they have no competing interests.

## Authors' contributions

WN participated in the design of the study and prepared manuscript. JH participated in the design of the study and prepared manuscript. CD participated in the design of the study, carried out the immunohistochemical assays, and prepared manuscript. RL participated in the design of the study and prepared manuscript. AG analyzed the data and prepared manuscript. All authors read and approved the final version of the manuscript.

## Pre-publication history

The pre-publication history for this paper can be accessed here:



## Figures and Tables

**Table 1:** Characteristic of 99 advanced serous epithelial ovarian carcinoma patients

**Characteristic**	**Number of cases(%)**
1. Age (years)	
≤ 20	2 (2.0)
20–30	3 (3.0)
31–40	3 (3.0)
41–50	25 (25.3)
51–60	36 (36.4)
61–70	23 (23.2)
71–80	7 (7.1)
2. Parity	
0	21 (21.2)
1	11 (11.1)
2	16 (16.2)
3	10 (10.1)
4	12 (12.1)
5	10 (10.1)
≥6	19 (19.2)
3. Chief complain	
Abdominal distension	54 (54.5)
Pelvic mass	33 (33.3)
Bleeding per vagina	4 (4.0)
Weight loss	1 (1.0)
Pelvic pain	7 (7.1)
4. FIGO Staging	
III	85 (85.9)
IV	14 (14.1)
5. Histologic grade	
1	60 (60.6)
2	24 (24.2)
3	15 (15.2)
6. Residual tumor (cm)	
None	8 (8.1)
>0–≤1	4 (4.0)
>1–≤2	50 (50.5)
>2	13 (13.2)
No data	24 (24.2)
7. Percent staining	
0	49 (49.5)
1–25	13 (13.1)
26–50	16 (16.2)
51–75	12 (12.1)
≥76	9 (9.1)
8. Intensity	
0 (no staining)	49 (49.5)
1+	29 (29.3)
2+	19 (19.2)
3+	2 (2.0)
9. Chemotherapy response	
Complete	62 (62.6)
Partial	8 (8.1)
Stable	8 (8.1)
Progressive	2 (21.2)
10. Recurrence	
Yes	54 (77.1)
No	16 (22.9)
11. Status at last follow-up	
DWD	69 (69.7)
DWID	4 (4.0)
AWD	3 (3.0)
ANED	7 (7.1)
Loss follow-up	16 (16.2)

DWD = death of disease, DWND = death with intercurrent disease, AWD = alive with Disease, ANED = alive with no evidence of disease

**Table 2:** Relationship of WT1 staining with other   clinicopathological variables of 99 advanced serous epithelial ovarian carcinoma   patients

**Characteristic**	**No staining (N = 49)**	**Staining (N = 50)**	**p-value**
1. FIGO Staging			0.967
III	42 (85.7)	43 (86.0)	
IV	7 (14.3)	7 (14.0)	
2. Histologic grade			0.610
1	32 (65.3)	28 (56.0)	
2	10 (20.4)	14 (28.0)	
3	7 (14.3)	8 (16.0)	
3. Residual tumor (cm)			0.009
None	8 (16.3)	5 (10.0)	
>0–≤1	2 (4.1)	6 (12.0)	
>1–≤2	3 (6.1)	1 (2.0)	
>2	18 (36.7)	32 (64.0)	
No data	18 (36.7)	6 (12.0)	
4. Chemotherapy response			0.036
Complete	37 (75.5)	25 (50.0)	
Partial	3 (6.1)	5 (10.0)	
Stable disease	4 (8.2)	4 (8.0)	
Progressive disease	5 (10.2)	16 (32.0)	

**Table 3:** Univariate analysis of potential prognostic factors for survival of 99 advanced serous epithelial ovarian carcinoma patients

**Characteristic**	**Hazard ratio**	**95%CI**	**p-value**
1. FIGO Staging			
III	1	-	0.6188
IV	1.21	0.58–2.56	
2. Histologic grade			
1	1	-	0.6569
2	1.31	0.74–2.30	
3	1.13	0.86–2.26	
3. Residual tumor (cm)			
None	1	-	0.3046
>0–≤1	2.24	0.79–6.37	
>1–≤2	0.99	0.21–4.62	
>2	1.75	0.87–3.66	
4. Chemotherapy response			
Complete	1	-	0.0001
Partial	0.99	0.39–2.53	
Stable	3.47	1.52–7.91	
Progressive	3.80	2.12–6.82	
5. WT1 staining			
No	1	-	0.0001
Yes	2.79	1.70–4.57	
6. Percent staining			
0	1	-	0.0008
1–25	4.05	1.89–8.70	
26–50	2.50	1.30–4.82	
51–75	1.97	0.86–4.54	
≥76	3.58	1.61–7.96	
7. Intensity			
0	1	-	0.0002
1+	2.56	1.47–4.48	
2+ or 3+	3.23	1.70–6.14	

**Table 4:** Multivariate analysis of potential prognostic factors for overall survival of 99 advanced serous epithelial ovarian carcinoma patients

**Characteristic**	**Hazard ratio**	**95%CI**	**p-value**
1. FIGO staging			
III	1	-	0.4602
IV	1.47	0.54 – 3.97	
2. Histologic grade			
1	1	-	0.9456
2	0.96	0.50 – 1.84	
3	1.11	0.50 – 2.46	
3. Residual tumor (cm)			
None	1	-	0.3522
>0 – 1	2.63	0.86 – 1.84	
>1 – 2	0.74	0.13 – 4.11	
>2	1.28	0.59 – 2.79	
4. Chemotherapy response			
Complete	1	-	0.0038
Partial	0.61	0.22 – 1.72	
Stable	2.29	0.86 – 6.13	
Progressive	2.93	1.53 – 5.62	
5. WT1 staining			
No	1	-	0.0138
Yes	1.98	1.28 – 3.79	

**Table 5:** Univariate analysis of potential prognostic factors for recurrence free survival among 62 advanced serous epithelial ovarian carcinoma patients showing complete response to chemotherapy

**Characteristic**	**Hazard ratio**	**95%CI**	**p-value**
1. FIGO Staging			
III	1	-	0.5904
IV	1.24	0.57–2.70	
2. Histologic grade			
1	1	-	0.9316
2	0.87	0.40–1.85	
3	0.98	0.45–2.14	
3. Residual tumor (cm)			
None	1	-	0.3123
>0–≤1	2.54	0.65–9.95	
>1–≤2	0.40	0.05–3.19	
>2	1.35	0.59–3.08	
4. WT1 staining			
No	1	-	0.0001
Yes	3.64	1.93–6.84	
5. Percent staining			
0	1	-	0.0002
1–50	5.22	2.51–10.82	
51–100	2.51	1.10–5.75	
6. Intensity			
0	1	-	0.0002
1+	3.11	1.55–6.24	
2+ or 3+	6.28	2.40–16.41	

**Table 6:** Multivariate analysis of potential prognostic factors for recurrence free survival among 62 advanced serous epithelial ovarian carcinoma patients showing complete response to chemotherapy

**Characteristic**	**Hazard ratio**	**95%CI**	**p-value**
1. FIGO staging			
III	1	-	0.8104
IV	1.14	0.40 – 3.22	
2. Histologic grade			
1	1	-	0.9783
2	0.93	0.41 – 2.10	
3	1.05	0.39 – 2.86	
3.Residual tumor (cm)			
None	1	-	0.8482
>0 – 1	0.93	0.21 – 4.13	
>1 – 2	0.41	0.05 – 3.55	
>2	0.88	0.36 – 2.16	
4.WT1 staining			
No	1	-	0.0017
Yes	3.36	1.60 – 7.03	

## References

[B1] Pataradool K, Korsiyatrakul T, Sriplung H, Sontipong S, Martin N, Wiangnon S, Vootiprux V, Cheirsilpa A (2003). Ovary. Cancer in Thailand 1995–1997.

[B2] Kritpracha K, Hanprasertpong J, Chandeying V, Dechsukhum C, Geater A (2005). Survival analysis in advanced epithelial ovarian carcinoma in relation to proliferative index of MIB-1 immunostaining. J Obstet Gynaecol Res.

[B3] Smith EM, Anderson B (1985). The effects of symptoms and delay in seeking diagnosis on stage of disease at diagnosis among women with cancers of the ovary. Cancer.

[B4] Landis SH, Murray T, Bolden S, Wingo PA (1999). Cancer statistics, 1999. CA Cancer J Clin.

[B5] Hartenbach EM, Olson TA, Goswitz JJ, Mohanraj D, Twiggs LB, Carson LF, Ramakrishnan S (1997). Vascular endothelial growth factor (VEGF) expression and survival in human epithelial ovarian carcinomas. Cancer Lett.

[B6] Daponte A, Guidozi F, Tiltman AJ, Marineanu A, Taylor L (1999). p53 as a prognostic factor for stage III serous adenocarcinoma of the ovary. Anticancer Res.

[B7] Ikeda K, Sakai K, Yamamoto R, Hareyama H, Tsumura N, Watari H, Shimizu M, Minakami H, Sakuragi N (2003). Multivariate analysis for prognostic significance of histologic subtype, GST-pi, MDR-1, and p53 in stages II–IV ovarian cancer. Int J Gynecol Cancer.

[B8] Milde-Langosch K, Hagen M, Bamberger AM, Loning T (2003). Expression and prognostic value of the cell-cycle regulatory proteins, Rb, p16MTS1, p21WAF1, p27KIP1, cyclin E, and cyclin D2, in ovarian cancer. Int J Gynecol Pathol.

[B9] Pritchard-Jones K, Fleming S, Davidson D, Bickmore W, Porteous D, Gosden C, Bard J, Buckler A, Pelletier J, Housman D, van Heyningen V, Hastie N (1990). The candidate Wilms'tumour gene is involved in genitourinary development. Nature.

[B10] Scharnhorst V, van der Eb AJ, Jochemsen AG (2001). WT1 proteins: functions in growth and differentiation. Gene.

[B11] Buckler AJ, Pelletier J, Haber DA, Glaser T, Housman DE (1991). Isolation, characterization, and expression of the murine Wilms' tumor gene (WT1) during kidney development. Mol Cell Biol.

[B12] Inoue K, Sugiyama H, Ogawa H, Nakagawa M, Yamagami T, Miwa H, Kita K, Hiraoka A, Masaoka T, Nasu K, Kyo T, Dohy H, Nakauchi H, Ishidate T, Akiyama T, Kishimoto T (1994). WT1 as a new prognostic factor and a new marker for the detection of minimal residual disease in acute leukemia. Blood.

[B13] Bergmann L, Miething C, Maurer U, Brieger J, Karakas T, Weidmann E, Hoelzer D (1997). High levels of Wilms' tumor gene (wt1) mRNA in acute myeloid leukemias are associated with a worse long-term outcome. Blood.

[B14] Ghanem MA, Van der Kwast TH, Den Hollander JC, Sudaryo MK, Oomen MH, Noordzij MA, Van den Heuvel MM, Nassef SM, Nijman RM, Van Steenbrugge GJ (2000). Expression and prognostic value of Wilms' tumor 1 and early growth response 1 proteins in nephroblastoma. Clin Cancer Res.

[B15] Miyoshi Y, Ando A, Egawa C, Taguchi T, Tamaki Y, Tamaki H, Sugiyama H, Noguchi S (2002). High expression of Wilms' tumor suppressor gene predicts poor prognosis in breast cancer patients. Clin Cancer Res.

[B16] Shimizu M, Toki T, Takagi Y, Konishi I, Fujii S (2000). Immunohistochemical detection of the Wilms' tumor gene (WT1) in epithelial ovarian tumors. Int J Gynecol Pathol.

[B17] Berek JS, Berek JS (2002). Ovarian cancer. Novak's Textbook of Gynecology.

[B18] Goldstein NS, Bassi D, Uzieblo A (2001). WT1 is an integral component of an antibody panel to distinguish pancreaticobiliary and some ovarian epithelial neoplasms. Am J Clin Pathol.

[B19] Hwang H, Quenneville L, Yaziji H, Gown AM (2004). Wilms tumor gene product: sensitive and contextually specific marker of serous carcinomas of ovarian surface epithelial origin. Appl Immunohistochem Mol Morphol.

[B20] WHO (1979). Handbook for reporting results of cancer treatment.

[B21] Schorge JO, Miller YB, Qi LJ, Muto MG, Welch WR, Berkowitz RS, Mok SC (2000). Genetic alterations of the WT1 gene in papillary serous carcinoma of the peritoneum. Gynecol Oncol.

[B22] Cohen HT, Bossone SA, Zhu G, McDonald GA, Sukhatme VP (1997). Sp1 is a critical regulator of the Wilms' tumor-1 gene. J Biol Chem.

[B23] McConnell MJ, Cunliffe HE, Chua LJ, Ward TA, Eccles MR (1997). Differential regulation of the human Wilms tumour suppressor gene (WT1) promoter by two isoforms of PAX2. Oncogene.

[B24] Fraizer GC, Shimamura R, Zhang X, Saunders GF (1997). PAX 8 regulates human WT1 transcription through a novel DNA binding site. J Biol Chem.

[B25] Dehbi M, Pelletier J (1996). PAX8-mediated activation of the wt1 tumor suppressor gene. EMBO J.

[B26] Discenza MT, Vaz D, Hassell JA, Pelletier J (2004). Activation of the WT1 tumor suppressor gene promoter by Pea3. FEBS Lett.

[B27] Maheswaran S, Englert C, Bennett P, Heinrich G, Haber DA (1995). The WT1 gene product stabilizes p53 and inhibits p53-mediated apoptosis. Genes Dev.

[B28] Johnstone RW, See RH, Sells SF, Wang J, Muthukkumar S, Englert C, Haber DA, Licht JD, Sugrue SP, Roberts T, Rangnekar VM, Shi Y (1996). A novel repressor, par-4, modulates transcription and growth suppression functions of the Wilms' tumor suppressor WT1. Mol Cell Biol.

[B29] Silberstein GB, Van Horn K, Strickland P, Roberts CT, Daniel CW (1997). Altered expression of the WT1 wilms tumor suppressor gene in human breast cancer. Proc Natl Acad Sci U S A.

[B30] Garg M, Moore H, Tobal K, Liu Yin JA (2003). Prognostic significance of quantitative analysis of WT1 gene transcripts by competitive reverse transcription polymerase chain reaction in acute leukaemia. Br J Haematol.

[B31] Hylander B, Repasky E, Shrikant P, Intengan M, Beck A, Driscoll D, Singhal P, Lele S, Odunsi K (2006). Expression of Wilms tumor gene (WT1) in epithelial ovarian cancer. Gynecol Oncol.

[B32] Oji Y, Ogawa H, Tamaki H, Oka Y, Tsuboi A, Kim EH, Soma T, Tatekawa T, Kawakami M, Asada M, Kishimoto T, Sugiyama H (1999). Expression of the Wilms' tumor gene WT1 in solid tumors and its involvement in tumor cell growth. Jpn J Cancer Res.

[B33] Omura GA, Brady MF, Homesley HD, Yordan E, Major FJ, Buchsbaum HJ, Park RC (1991). Long-term follow-up and prognostic factor analysis in advanced ovarian carcinoma:the Gynecologic Oncology Group experience. J Clin Oncol.

[B34] Brinkhuis M, Meijer GA, Baak JP (1995). An evaluation of prognostic factors in advanced ovarian cancer. Eur J Obstet Gynecol Reprod Biol.

